# Association of the Controlling Nutritional Status Score with the Development of Postoperative Paralytic Ileus After Radical Cystectomy: Retrospective Cohort Study

**DOI:** 10.5152/tud.2023.22232

**Published:** 2023-05-01

**Authors:** Kenji Tanabe, Yasukazu Nakanishi, Noya Okubo, Yousuke Umino, Madoka Kataoka, Shugo Yajima, Hitoshi Masuda

**Affiliations:** Department of Urology, National Cancer Center Hospital East, Chiba, Japan

**Keywords:** Urinary bladder neoplasms, nutritional status, ileus, cystectomy

## Abstract

**Objective:**

Postoperative paralytic ileus is a major adverse event of radical cystectomy, causing prolonged hospitalization. The controlling nutritional status score, consisting of serum albumin, total lymphocyte count, and total cholesterol, indicates the nutritional status and may evaluate gastrointestinal status. This study aimed to clarify the association between the controlling nutritional status score and the development of postoperative paralytic ileus in patients who underwent radical cystectomy with ileal conduit or ileal neobladder.

**Materials and Methods:**

We retrospectively analyzed the clinical features of patients who underwent open radical cystectomy or robotic assisted laparoscopic radical cystectomy with ileal conduit or ileal neobladder for bladder cancer between April 2011 and May 2021. The association between clinical variables, including the controlling nutritional status score and the development of postoperative paralytic ileus, was examined.

**Results:**

Out of 133 patients, 34 (26%) developed postoperative paralytic ileus. The patients who developed postoperative paralytic ileus were likely to have a higher controlling nutritional status score (*P* = .055) compared to those who did not develop postoperative paralytic ileus. Multivariate analysis revealed that a preoperative controlling nutritional status score of ≥1 (odds ratio: 2.90, 95% CI: 1.08-7.80, *P* = .034) and longer operating time (odds ratio: 3.02, 95% CI: 1.13-8.11, *P* = .027) were significant independent factors for postoperative paralytic ileus development.

**Conclusion:**

A high controlling nutritional status score and long operating time may be risk factors for developing postoperative paralytic ileus in patients who underwent radical cystectomy with ileal conduit or ileal neobladder for bladder cancer. Preoperative controlling nutritional status may be able to predict postoperative paralytic ileus development.

**Main Points**

- No previous studies have analyzed the association between the controlling nutritional status (CONUT) score, a tool of nutritional assessment, and the development of postoperative paralytic ileus (POI) after radical cystectomy.
- This is the first study to identify a higher CONUT score as a risk factor for developing POI (odds ratio: 2.90, 95% CI: 1.08-7.80, *P* = .034).
- Preoperative CONUT score may be able to predict POI development.

## Introduction

The outcomes of radical cystectomy have improved, decreasing its perioperative mortality rate. On the other hand, the incidence of postoperative adverse events remains high, ranging from 25% to 64%.^1,2^ Therefore, radical cystectomy is associated with the highest hospitalization costs and longest hospital stays for patients undergoing urological surgery.^3^

Postoperative paralytic ileus (POI) is a major adverse event of radical cystectomy that causes prolonged hospitalization. Postoperative paralytic ileus occurs in 10%-40% of patients undergoing radical cystectomy, accounting for 50%-70% of all complications in radical cystectomy.^2,3^ Age, body mass index (BMI), the estimated amount of blood loss during surgery, electrolyte abnormalities, and use of epidural anesthesia are reportedly POI risk factors, but there is no consensus.^1,4-6^ It is commonly known that after major abdominal surgery, there is a temporary decrease in the contractile activity of the bowel. There is a known time lag in recovering contractile activity in the stomach, small intestine, and large intestine.^3^ This time lag can lead to long-term inhibition of intestinal contractile activity, with secretions and gas, nausea, vomiting, and gastric distention, which appear to cause POI.^3^

In recent years, the controlling nutritional status (CONUT) score, which consists of serum albumin level, total lymphocyte count, and total cholesterol level,^7^ is reportedly a useful predictor of survival in advanced cancer, including urological cancer, and perioperative complications.^8-12^ Since the CONUT score can detect minor nutritional decline,^7^ it could be used to evaluate gastrointestinal status after surgical intervention.

This study aimed to clarify the association between CONUT score and POI development. It identified POI risk factors in patients who underwent radical cystectomy with ileal conduit or ileal neobladder for bladder cancer.

## Materials and Methods

### Patients

We retrospectively analyzed the clinical data of consecutive patients who underwent open radical cystectomy (ORC) or robotic assisted laparoscopic radical cystectomy (RARC) with ileal conduit or ileal neobladder for bladder cancer at our institution between April 2011 and May 2021. Two patients preoperatively diagnosed with distant metastases were excluded from the study, resulting in a total of 133 patients. Out of the 133 cases, 86 underwent ORC and 47 underwent RARC. Among the RARC cases, 22 underwent intracorporeal urinary diversion (ICUD), and 25 patients underwent extracorporeal urinary diversion (ECUD). All patients underwent transurethral resection of the bladder tumor prior to radical cystectomy and were diagnosed with bladder cancer. Basically, 4 cycles of cisplatin-based chemotherapy were administered as a neoadjuvant treatment for muscle-invasive bladder cancer patients with chemotherapy-complement conditions.

### Surgical Procedure

Patients underwent radical cystectomy with bilateral pelvic lymphadenectomy to the level of common iliac arteries whenever possible, followed by ECUD. The resection lengths of the terminal ileum were 20 cm and 55 cm for the ileal conduit and neobladder, respectively. In ORC and RARC (including both ECUD and ICUD) cases, intestinal anastomosis was performed by functional end-to-end anastomosis with laparotomy. The same technique of intestinal anastomosis was used for the patients with neobladder reconstruction. All ICUD cases were performed ileal conduit. Open radical cystectomy was performed by 6 surgeons, 5 of whom were urologists and 1 was a resident. Robotic assisted laparoscopic radical cystectomy was performed by 3 surgeons, 2 of whom were novice surgeons with <20 RARCs. The 2 surgeons performed the procedure under the guidance of a senior surgeon (H.M.). Since the introduction of RARC, patients who were expected to have difficulty performing RARC, such as those with a history of pelvic surgery, were indicated for ORC.

### Perioperative Management

All patients were on the same clinical pathway. Patients consumed normal meals orally until the day before surgery and took stimulant laxatives internally the night before surgery. Intraoperatively, all patients received prophylactic antimicrobial agents (second-generation cephem based), and the ileum used for urinary derivations was thoroughly washed with warm saline. Epidural anesthesia with opioids was used for patients when indicated, and intravenous patient-controlled analgesia with opioids was used for the rest of the patients. Intraoperatively, intestinal decompression was performed with a gastric tube removed on the first postoperative day. Postoperatively, blood tests and abdominal x-rays were basically performed on the day after surgery, the third day after surgery, and the fifth day after surgery and thereafter according to the patient’s condition. Ambulation was encouraged as much as possible on the first postoperative day. For patients undergoing epidural anesthesia, it was completed 3 days after surgery. For patients undergoing intravenous patient-controlled analgesia (IV-PCA), it was completed 2 days after surgery. Abdominal x-rays were taken on the first, third, and fifth postoperative days. Patients began drinking water on the first postoperative day, and the physician in charge determined the diet based on the patient’s general condition and abdominal x-ray findings. Intestinal motility stimulants were not routinely used.

The development of POI was determined based on the clinical and imaging findings, as defined by Vather et al^13^ and Maglinte et al.^14^ Vather et al defined prolonged postoperative ileus as having 2 or more of the following criteria: A. Nausea or vomiting, B. Inability to tolerate an oral diet over the last 24 hours, C. Absence of flatus over last 24 hours, D. Abdominal distension, and E. Radiologic confirmation. Regarding the criterion E, Maglinte et al used the findings of abnormal gaseous or fluid distension of small intestine and air–fluid levels as the definition of radiographic findings of POI. We defined POI as a patient who has one of the criteria A-D and has criterion E. We assessed POI using Clavien–Dindo classification (CDC) of surgical complications (version 2.0).^15^ Once the patient could begin oral intake, the above condition was defined as the development of POI. The above condition was defined as the development of POI. Postoperative paralytic ileus with CDC ≤ II was treated with stopping the diet and use of intestinal peristalsis stimulants, while all 9 patients with CDC IIIa were treated by the insertion of the ileus tube. Some of the patients who developed postoperative ileus required total parenteral nutrition.

### Data Collection

The following variables were analyzed: age, sex, BMI, Eastern Cooperative Oncology Group performance status, American Society of Anesthesiology physical status, smoking history, history of abdominal surgery, history of abdominal radiotherapy, clinical staging, presence of neoadjuvant chemotherapy, presence of preoperative anemia, presence of preoperative renal dysfunction, preoperative CONUT score, urinary derivation method, use of epidural anesthesia, operating time, the estimated amount of blood loss (EBL), presence of perioperative blood transfusion, and several serum electrolyte levels. The 8th edition of the International Union Against Cancer was used for preoperative staging. Blood samples were collected approximately 1 month before the date of surgery and the day before surgery. Anemia was defined as hemoglobin <13.0 g/dL in men and 12.0 g/dL in women. Renal dysfunction was defined as an estimated glomerular filtration rate of less than 60 mL/min/1.73 m^2^. Hyponatremia was defined as a serum sodium <135 mEq/L. Hypochloremia was defined as a serum chloride <96 mEq/L. Hypokalemia was defined as a serum potassium <3.5 mEq/L. The CONUT score was calculated by the method shown in Table 1 which was a categorical variable. All variables were classified into 2 groups.^2^ Among the continuous variables, the cutoff values for age and BMI were adopted as general values.^8^ The cutoff values for operating time and estimated blood loss were determined by calculating the maximum Youden index through the receiver operating characteristic (ROC) curve analysis, with the development of POI as the endpoint. The CONUT score was also subjected to ROC curve analysis in the same way as continuous variables, and the maximum Youden index was calculated as the cutoff value. The primary endpoint was the development of POI within 1 month after surgery.

### Statistical Analysis

The differences between groups were analyzed using the chi-square test or Fisher’s exact test for categorical variables and the Wilcoxon–Mann–Whitney *U*-test for continuous data. Univariate and multivariate logistic regression analyses were used to evaluate the association between clinical and operative characteristics and the development of POI. A reduced multivariate model was based on the stepwise backward method, in which the variable with the highest *P*-value was eliminated from each iteration of the multivariate analysis. Statistical significance was set at *P* values less than .05. All statistical analyses were performed using the JMP software (SAS Institute Inc., Cary, NC, USA, version 13.2).

### Ethical Standards and Policies

The institution’s Ethics Committee approved the protocol for this research project, and it complied with the provisions of the Declaration of Helsinki (National Cancer Center Research Ethics Review Committee, Research Project No. 2018-159). An opt-out method was applied to obtain consent for this retrospective study.

## Results

Table 2 shows the clinical and operative characteristics of the patients grouped according to developing POI or not. The median age was 71 years (interquartile range 65-76 years), and 34 patients (26%) developed POI. The patients who developed POI were likely to have a higher CONUT score (*P* = .055) compared to those that did not develop POI, although the absence of differences was not significant. Among 34 patients who developed POI, 25 and 9 patients were classified as CDC Grade II and IIIa, respectively.

Table 3 shows the associations between patients and operative characteristics and POI and the cutoff values of variables, including CONUT score. The cutoff value for CNOUT score was calculated to be 1 with AUC values of 0.58. The cutoff values for operative time and EBL by calculating the maximum Youden Index through the ROC curve analysis were 404 minutes and 742 g with AUC values of 0.59 and 0.55, respectively. On multivariate analysis, a preoperative CONUT score of ≥1 (odds ratio: OR 2.90, 95% confidence interval: CI 1.08–7.80, *P*= 0.034) and longer operating time of ≥404 minutes (OR 3.03, 95% CI 1.13–8.11, *P*= 0.027) significantly were independent factors that significantly affected the development of POI.

## Discussion

In this study cohort, 26% of patients who underwent ORC or RARC with ileal conduit or ileal neobladder for bladder cancer developed POI. This is the first report to show that the preoperative CONUT score is an independent risk factor for the development of POI. This study examined a combined cohort of ORC and RARC procedures. According to a meta-analysis that evaluated the outcomes between ORC and RARC, there was no difference in the incidence of complications.^16^ Moreover, there is currently no substantial evidence regarding the incidence of POI after radical cystectomy in the combined cohort of both groups. The incidence of POI in our study was higher than that reported by other authors,^4,17^ but this may be because the use of epidural anesthesia was very high (90%), and we enrolled patients who underwent radical cystectomy with ileal conduit or ileal neobladder only and did not enroll patients with ureterocutaneous fistulas.

Even though POI is a major adverse event that develops in patients undergoing major abdominal surgery, there has been no significant progress in understanding or treating it. The definition of POI also differs among reports, with the recovery of bowel peristalsis sounds and exhaust gas often used as indicators.^3,4,18^ However, these are only subjective judgments made. In this study, vomiting (an obvious clinical symptom) and abdominal x-ray images (which are objective findings), were used to define POI development. In previous reports, the use of epidural anesthesia, EBL, and age were risk factors for POI development.^4,19,20^ However, none of these factors was associated with POI in our cohort. The use of epidural anesthesia was very high in our cohort (90%), which may have led to different results from other studies.

This is the first study to identify a higher CONUT score as a risk factor for developing POI. The CONUT score is a tool developed in 2003 to assess hospitalized patients’ nutritional status.^7^ Easily, it was a simple, low-cost, and efficient tool that requires only 3 blood test results. Recently, the CONUT score was reportedly a good predictor of perioperative complications and cancer prognosis in several carcinomas.^8-11^ In urology, several reports have shown that the group with a high CONUT score had significantly higher perioperative pulmonary complications and poorer cancer prognosis.^8,11^ However, there have been no reports on the association of CONUT score with POI development. In the current study, a CONUT score of 1 was the cutoff value that significantly affected the perioperative period. A CONUT score of ≥2 was originally defined as nutritional impairment and a CONUT score of 1 was within the normal range.^7^ However, in the perioperative period, a seemingly minor decrease in nutrition can lead to intestinal complications that would be clinically evident. Traditionally, there is a long fasting period in the perioperative period of major abdominal surgery. However, a decrease in total protein and serum albumin levels due to low nutrition caused by this long period of fasting increases the risk of serious adverse postoperative events, such as delayed wound healing and postoperative infection.^17^ Although in the field of colorectal surgery, in a similar report in surgery involving intestinal manipulation as in the present study, Dai et al^16^ revealed that preoperative hypoalbuminemia is an independent risk factor for POI development after colorectal resection. ここをクリックまたはタップしてテキストを入力してください。 They also discussed that low nutritional status could lead to intestinal edema, which triggers the development of POI. Improving preoperative nutritional status can help prevent POI development. Therefore, the practice of perioperative fasting is currently being reevaluated. In our cohort, there was no long fasting period before surgery, and the resumption of eating was not delayed unnecessarily. However, nutritional status generally declines after surgery due to various factors, such as stress from a surgical invasion, dilution from postoperative fluids, and the time it takes to resume eating.^21^ This study’s results may suggest that patients with a preoperative tendency to be undernourished may be at higher risk of developing POI as an adverse event due to further decline in nutritional status after surgery. Although Nemoto et al^8^ and Suzuki et al^12^ showed that the CONUT score was related to oncologic prognosis in advanced bladder cancer, the present study found no relationship between the CONUT score and oncologic prognosis. The cutoff value for the CONUT score was set at 3 or 2 in the previous reports. The fact that this study included only localized bladder cancer and the cutoff value of the CONUT score was different may be the reason for the difference between the previous reports and this study.

There are reports on the relationship between operating time and POI development.^18,22^ Several theories for the mechanism of POI after abdominal surgery have also been proposed. The sympathetic nervous system disturbance caused by surgery, the collection of inflammatory cells and inflammatory substances in the intestinal tract, and the side effects of anesthetics have all been attributed to POI. The interaction of these factors is also thought to be a cause of POI.^23^ These effects could become stronger as the operative time increases. A longer operation time for the same procedure may also reflect a more difficult operation, in which the effects above will likely be stronger. More studies are expected on the relationship between operating time and POI development.

There are several limitations to this study. First, this study is a retrospective study with inherent limitations. Second, some aspects of postoperative management may be left to the treating physician’s discretion and may not be uniform. However, the same clinical pathway was used for all patients, minimizing the variability in management based on the treating physician’s judgment. Third, the number of cases is small. We calculated the number of cases needed in this study using G*power software (version: 3.1.9.6) by considering a significant level of 0.05 and power of 80% for detecting a medium effect size (d = 0.48) under the chi-square test. By considering these settings, 48 cases for the total sample size were calculated, confirming that the number of cases in this study was sufficient. Furthermore, the definition of the development of POI in this study differs from the definition reported by other authors.^3,4,18^ In most reports, postoperative bowel obstruction is defined as delayed recovery of bowel function, which is often judged by the presence or absence of bowel peristalsis or gas. However, this is based on the subjectivity of the observer and patient. In this study, abdominal x-ray examination was routinely performed across all patients, and this objective finding defined POI, which is a unique point of this study.

## Conclusion

Preoperative CONUT score (an indicator of nutritional status) and operating time were independent risk factors associated with POI development in patients undergoing radical cystectomy ileal conduit or ileal neobladder for bladder cancer. Preoperative CONUT may be able to predict POI development.

## Figures and Tables

**Table 1.** Calculation of the Controlling Nutritional Status Score

**Parameter**				
Serum albumin, g/dL	≥3.5	3.00-3.49	2.50-2.99	<2.50
Score	0	2	4	6
Total lymphocyte count, /mm^3^	≥1600	1200-1599	800-1199	<800
Score	0	1	2	3
Total cholesterol, mg/dL	≥180	140-179	100-139	<100
Score	0	1	2	3

**Table 2.** Patients and Operative Characteristics by the Surgical Method

Variable	Total (n = 133)	POI (n = 34)	Non-POI (n = 99)	*P*
Age (years), median (IQR)	71 (65-76)	70 (64-73)	71 (65-76)	.72
Sex, n (%)				1.00
Male	107 (80)	27 (79)	80 (81)	
Female	26 (29)	7 (21)	19 (19)	
BMI (kg/m^2^), median (IQR)	23.6 (21.4-25.0)	23.8 (19.7-25.9)	23.6 (21.8-24.8)	.55
ECOG PS, n (%)				.42
0	109 (82)	26 (76)	83 (84)	
1	23 (17)	8 (24)	15 (15)	
2	1 (1)	0 (0)	1 (1)	
ASA PS, n (%)				.32
1	49 (37)	15 (44)	34 (34)	
2	73 (55)	15 (44)	58 (59)	
3	11 (8)	4 (12)	7 (7)	
Smoking, n (%)				.52
No	25 (19)	8 (23)	17 (17)	
Yes	106 (80)	25 (74)	81 (82)	
Unknown	2 (1)	1 (3)	1 (1)	
Abdominal surgery, n (%)				.69
No	73 (55)	20 (59)	53 (54)	
Yes	60 (45)	14 (41)	46 (46)	
Abdominal radiotherapy, n (%)				1.00
No	129 (97)	33 (97)	96 (97)	
Yes	4 (3)	1 (3)	3 (3)	
Clinical T stage, n (%)				.51
a or 1 or is	36 (27)	10 (29)	26 (26)	
2	44 (33)	14 (41)	30 (30)	
3	41 (31)	8 (24)	33 (34)	
4	12 (9)	2 (6)	10 (10)	
Clinical N stage, n (%)				.92
0	111 (83)	28 (82)	83 (84)	
1	17 (13)	5 (15)	12 (12)	
2	4 (3)	1 (3)	3 (3)	
3	1 (1)	0 (0)	1 (1)	
NAC, n (%)				.31
No	59 (45)	18 (53)	41 (42)	
Yes	74 (55)	16 (47)	57 (58)	
Preoperative anemia^*^, n (%)				.31
No	52 (39)	16 (47)	36 (36)	
Yes	81 (61)	18 (53)	63 (64)	
Preoperative renal dysfunction^†^, n (%)				.42
No	76 (57)	17 (50)	59 (60)	
Yes	57 (43)	17 (50)	40 (40)	
Preoperative sodium (mEq/L), median (IQR)	142 (140-143)	141 (139-142)	142 (141-143)	.081
Preoperative chloride (mEq/L), median (IQR)	105 (103-107)	105 (103-106)	105 (103-107)	.88
Preoperative potassium (mEq/L), median (IQR)	4.0 (3.7-4.2)	4.3 (4.1-4.4)	4.3 (4.0-4.5)	.83
CONUT score, n (%)				.055
0	43 (32)	6 (18)	37 (38)	
1	45 (34)	15 (44)	30 (30)	
2-4	44 (33)	12 (35)	32 (32)	
5-8	1 (1)	1 (3)	0 (0)	
Urinary derivation method, n (%)				.78
Ileal conduit	114 (86)	30 (88)	84 (85)	
Neobladder	19 (14)	4 (12)	15 (15)	
Epidural anesthesia, n (%)				.73
No	13 (10)	4 (12)	9 (9)	
Yes	120 (90)	30 (88)	90 (91)	
Operating time (minutes), median (IQR)	429 (387-491)	426 (405-494)	431 (384-490)	.28
EBL (g), median (IQR)	710 (387-1282)	854 (446-1623)	673 (372-1178)	.17
POD1 sodium (mEq/L), median (IQR)	141 (139-142)	140 (139-143)	141 (139-142)	.91
POD3 sodium (mEq/L), median (IQR)	139 (136-141)	138 (136-141)	139 (137-141)	.46
POD1 chloride (mEq/L), median (IQR)	107 (106-109)	107 (106-109)	107 (105-109)	.48
POD3 chloride (mEq/L), median (IQR)	104 (102-106)	104 (101-107)	104 (102-106)	.68
POD1 potassium (mEq/L), median (IQR)	4.0 (3.8-4.2)	4.0 (3.7-4.2)	3.9 (3.8-4.2)	.89
POD3 potassium (mEq/L), median (IQR)	4.3 (4.0-4.5)	4.0 (3.9-4.2)	4.0 (3.8-4.2)	.46

ASA PS, American Society of Anesthesiology physical status; BMI, body mass index; CONUT, controlling nutritional status; EBL, estimated amount of blood loss; ECOG PS, Eastern Cooperative Oncology Group performance status; IQR, interquartile range; NAC, neoadjuvant chemotherapy; POD, postoperative day; POI, postoperative paralytic ileus.

^*^Anemia is defined as hemoglobin levels of <13.5 g/dL and <12.0 g/dL in males and females, respectively.

^†^Renal dysfunction is defined as an estimated glomerular filtration rate of <60 mL/min/1.73 m^2^.

**Table 3.** Univariate and Multivariate Logistic Regression Analyses of Association Between Patients and Operative Characteristics with Postoperative Paralytic Ileus

Variable	Category	Univariate Analysis			Multivariate Analysis		
		OR	95% CI	*P*	OR	95% CI	*P*
Age (years)	≥70 vs. <70	1.01	0.45-2.22	.98			
Sex	Men vs. women	0.92	0.35-2.42	.86			
BMI (kg/m^2^)	≥25 vs. <25	1.80	0.77-4.19	.17			
ECOG PS	≥1 vs. 0	1.60	0.61-4.15	.35			
ASA PS	≥3 vs. 1-2	1.75	0.48-6.40	.41			
Smoking	Yes vs. no	0.65	0.25-1.70	.39			
Abdominal surgery	Yes vs. no	0.81	0.37-1.78	.59			
Abdominal radiotherapy	Yes vs. no	0.97	0.10-9.65	.98			
Clinical T stage	≥3 vs. ≤2	0.51	0.22-1.20	.11			
Clinical N stage	≥1 vs. 0	1.11	0.40-3.11	.84			
NAC	Yes vs. no	0.64	0.29-1.40	.26			
Preoperative anemia	Yes vs. no	0.64	0.29-1.41	.27			
Preoperative renal dysfunction	Yes vs. no	1.48	0.67-3.23	.33			
Preoperative hyponatremia*	Yes vs. no	N/A	N/A	N/A			
Preoperative hypochloremia^†^	Yes vs. no	N/A	N/A	N/A			
Preoperative hypokalemia^‡^	Yes vs. no	N/A	N/A	N/A			
CONUT score	≥1 vs. 0	2.78	1.05-7.36	.028	2.91	1.08-7.80	.034
Surgical method	Open vs. robot-assisted	1.00	0.44-2.25	1.00			
Urinary derivation method	Ileal conduct vs. neobladder	0.75	0.23-2.43	.62			
Epidural anesthesia	Yes vs. no	0.75	0.22-2.61	.66			
Operating time^||^ (min)	≥404 vs. <404	2.91	1.10-7.67	.021	3.03	1.13-8.11	.027
EBL^||^ (g)	≥742 vs. <742	2.02	0.91-4.48	.080			
Blood transfusion	Yes vs. no	1.41	0.64-3.07	.39			
POD1 hyponatremia^†^	Yes vs. no	N/A	N/A	N/A			
POD3 hyponatremia^†^	Yes vs. no	0.62	0.12-3.04	.56			
POD1 hypochloremia^‡^	Yes vs. no	N/A	N/A	N/A			
POD3 hypochloremia^‡^	Yes vs. no	N/A	N/A	N/A			
POD1 hypokalemia^§^	Yes vs. no	1.81	0.41-8.05	.43			
POD3 hypokalemia^§^	Yes vs. no	0.96	0.097-9.64	.97			

ASA PS, American Society of Anesthesiology physical status; BMI, body mass index; CONUT, controlling nutritional status; EBL, estimated amount of blood loss; ECOG PS, Eastern Cooperative Oncology Group performance status; N/A, not available; NAC, neoadjuvant chemotherapy; OR, odds ratio; POD, postoperative day.

^*^Hyponatremia was defined as a serum sodium <135 mEq/L.

^†^Hypochloremia was defined as a serum chloride <96 mEq/L.

^‡^Hypokalemia was defined as a serum potassium <3.5 mEq/L.

^§^Hypocalcemia was defined as a serum calcium <8.5 mg/dL.

^||^The cutoff values for operating time and EBL were determined by calculating the maximum Youden index through the receiver operating characteristic curve analysis, with the development of POI as the endpoint.
